# Green mycosynthesis of a CuO/ZnO heterojunction nanocomposite using *Aspergillus terreus* and its antibacterial and anti-virulence activity against multidrug-resistant *Escherichia coli*

**DOI:** 10.1038/s41598-026-44775-z

**Published:** 2026-04-13

**Authors:** Ahmed Nafia Obaid, Tarek M. Abdelghany, Amal M. Soliman, Nashaat N. Mahmoud, Alsayed E. Mekky

**Affiliations:** 1 Botany and Microbiology Department, Faculty of Science, Al-Azhar University, Nasr City, Cairo, 11884 Egypt; 2 Medical Microbiology and Immunology Department, Faculty of Medicine, Ain Shams University, Cairo, Egypt

**Keywords:** Multidrug-resistant *Escherichia coli*, CuO/ZnO nanocomposite, *Aspergillus terreus*, Antibacterial activity, Quorum sensing inhibition, Virulence genes, Biochemistry, Biological techniques, Biotechnology, Microbiology

## Abstract

The rapid spread of multidrug-resistant (MDR) *Escherichia coli* poses a severe global health risk and demands the development of new antibacterial strategies. Biologically manufactured metal oxide nanocomposites have gained popularity due to their superior antibacterial activity and environmentally sustainable synthesis. This study uniquely integrates fungal metabolite profiling, green synthesis of a CuO/ZnO heterojunction nanocomposite, and molecular evaluation of anti-virulence activity against clinically isolated MDR *Escherichia coli*. The CuO/ZnO system was selected due to its synergistic heterostructure, which enhances reactive oxygen species generation and improves antibacterial performance compared to single metal oxides. In this investigation, an MDR *E. coli* strain was isolated from wound infections and identified using 16 S rRNA sequencing. A soil-derived fungus capable of forming CuO/ZnO nanocomposites was isolated and identified as *Aspergillus terreus* using 18 S rRNA sequencing. The bioactive constituents in the fungal filtrate were identified using High performance liquid chromatography (HPLC) and Gas chromatography-mass spectrometry (GC-MS). UV-Vis spectroscopy, transmission electron microscope (TEM), Fourier transform infrared spectroscopy(FTIR), and X-ray diffraction (XRD) were used to characterize the biosynthesized CuO/ZnO nanocomposite. The antibacterial effect against MDR *E. coli* was examined using disc diffusion and minimum inhibitory concentration (MIC) assays, and the influence on virulence and quorum-sensing genes was measured using quantitative reverse transcription PCR (qRT-PCR). The MDR *E. coli* isolate was resistant to all antibiotics tested. A variety of phenolic acids, flavonoids, and aromatic compounds were detected in the fungal filtrate; however, the filtrate alone did not exhibit antibacterial activity. The CuO/ZnO nanocomposite showed strong antibacterial activity, with an inhibition zone diameter of 2.3 ± 0.4 mm and MIC of 62.5 ± 0.2 µg/mL. Gene expression analysis indicated considerable downregulation of critical virulence genes, including *fimH*, *luxS*, *toxA*, and *papC*, suggesting impairment of adhesion, toxin synthesis, and quorum sensing. The biosynthesized CuO/ZnO nanocomposite shows strong antibacterial and anti-virulence activity against MDR *E. coli*, indicating its potential as an alternative antibacterial agent.

**Supplementary Information:**

The online version contains supplementary material available at 10.1038/s41598-026-44775-z.

## Introduction

Microbial infections remain to be one of the biggest threats to global public health, despite substantial advances in medical research and antibiotic therapy. The rising incidence of antibiotic-resistant pathogenic bacteria has hampered infection management, resulting in increased morbidity, mortality, and healthcare costs^1,2^. *Escherichia coli* is a well-known bacterial pathogen due to its widespread distribution, its capacity to develop resistance and virulence factors, and involvement in a variety of illnesses, including gastrointestinal, urinary tract, and systemic infections^3^. In developing nations, such as Iraq, pathogenic *E. coli* infections create a significant clinical burden, highlighting the urgent need for innovative and effective antimicrobial treatments^4,5^.

Numerous microorganisms, particularly fungal species and probiotics, have long been known to produce a wide range of bioactive compounds^6^. Filamentous fungi of the genus *Aspergillus* are particularly interesting due to their metabolic complexity and tolerance to various ecological niches. Among *Aspergillus* species, *Aspergillus terreus* was specifically selected for this study due to its well-documented capacity to produce diverse secondary metabolites, including phenolic acids, flavonoids, polyketides, and reductive biomolecules, which can function as natural reducing and stabilizing agents during nanoparticle synthesis^7^. In addition, *A. terreus* has demonstrated high metabolic adaptability, efficient extracellular enzyme secretion, and previous success in biosynthesizing metal and metal oxide nanoparticles with controlled size and enhanced stability. These characteristics make it a particularly suitable candidate for the eco-friendly fabrication of metal oxide nanocomposites with improved biological functionality^7,8^. Accurate identification of fungal isolates using both classical phenotypic criteria and genetic approaches is critical for determining the functional capability and biotechnological potential of specific species^9,10^. The use of a freshly isolated fungal strain in the present study was intentional, as environmental isolates often exhibit enhanced metabolic activity, adaptive stress tolerance, and more diverse secondary metabolite profiles compared to long-maintained laboratory strains. Fresh isolates may secrete higher levels of reductive biomolecules and extracellular enzymes, which are critical for efficient metal ion reduction and nanoparticle stabilization during green synthesis^9,10^. Therefore, employing a newly isolated *A. terreus* strain was expected to improve biosynthetic efficiency and nanocomposite functionality.

In recent years, the development of environmentally friendly nanotechnology has gained prominence as a promising strategy for combating microbial resistance^11^. The manufacturing of green nanoparticles (NPs) using biological systems offers several advantages over traditional physical and chemical approaches, including decreased toxicity, lower energy consumption, and improved biocompatibility^12^. Fungal-mediated production of metal and metal oxide NPs is gaining popularity due to fungi’s capacity to release large amounts of enzymes and metabolites that act as reducing and stabilizing agents^13,14^. These biological constituents not only promote nanoparticle production but may also enhance antibacterial activity through surface functionalization^15^. Copper oxide (CuO) and zinc oxide (ZnO) nanoparticles have attracted significant attention due to their unique physicochemical properties, including high surface area-to-volume ratio, semiconducting behavior, strong redox activity, and intrinsic antimicrobial potential. Beyond antibacterial applications, CuO and ZnO nanomaterials have been widely explored in environmental remediation, photocatalysis, sensing technologies, energy storage systems, anticorrosion coatings, and biomedical applications owing to their catalytic efficiency and cost-effectiveness^16–19^. ZnO nanoparticles, in particular, exhibit excellent UV-blocking capability and biocompatibility, while CuO nanoparticles demonstrate superior redox cycling ability and enhanced reactive oxygen species generation, making them highly effective in oxidative degradation and antimicrobial systems^16,18^. Furthermore, recent advances highlight the development of CuO- and ZnO-based composite materials for improved charge separation efficiency and enhanced multifunctional performance in catalytic and biomedical fields^17,20^. These diverse applications and synergistic physicochemical properties justify the continued investigation of CuO/ZnO nanostructures for advanced antimicrobial strategies^16,18,20^.

Copper oxide (CuO) and zinc oxide (ZnO) are metal oxides that, when engineered at the nanoscale, exhibit significant antibacterial activity against a wide range of microorganisms. When combined as a nanocomposite, CuO/ZnO exhibits synergistic effects that enhance its antibacterial properties through various mechanisms, including disruption of bacterial cell membranes, generation of reactive oxygen species, and interference with key metabolic pathways^21^. The incorporation of fungal bioactive compounds into nanocomposite synthesis enhances their robustness and bioactivity, making these systems promising candidates for antibacterial applications^22^. Although several metal nanoparticles, including silver (Ag), gold (Au), and titanium dioxide (TiO₂), have demonstrated strong antibacterial activity, their clinical and environmental applications may be limited by cost, potential cytotoxicity, and stability concerns^20,23^. In contrast, copper oxide (CuO) and zinc oxide (ZnO) nanoparticles are comparatively cost-effective, exhibit broad-spectrum antimicrobial properties, and possess acceptable biocompatibility profiles^21,24^. Importantly, combining CuO and ZnO into a heterojunction nanocomposite enhances charge separation efficiency and reactive oxygen species (ROS) generation, leading to synergistically improved antibacterial performance compared to single metal oxides^24–26^. Therefore, the selection of a CuO/ZnO nanocomposite in the present study was based on its dual-metal synergistic mechanism, economic feasibility, enhanced oxidative antibacterial potential, and suitability for green biosynthesis approaches.

Understanding bacterial pathogenicity requires not only phenotypic evaluation but also molecular analysis of virulence and communication mechanisms. The pathogenicity of *E. coli* is intimately linked to genes associated with adhesion (*fimH*, *papC*), toxin synthesis (*toxA*), and quorum sensing (*luxS*), all of which affect colonization, survival, and infection development^27^. Molecular identification and gene monitoring using housekeeping genes such as 16 S rRNA are reliable approaches for accurately detecting and characterizing pathogenic organisms. Furthermore, assessing how novel antimicrobial agents affect the expression of these virulence-related genes provides insight about their ability to reduce bacterial pathogenicity beyond mere growth inhibition^28^.

Given the growing problem of antimicrobial resistance and the limited efficacy of current antibiotics, there is increasing interest in investigating integrated biological and nanotechnology-based approaches. The combination of fungal biotechnology, bioactive metabolite profiling, nanocomposite synthesis, and molecular microbiology offers a comprehensive strategy for developing novel antimicrobial agents^29^. Such approaches are especially important in regions like Iraq, where bacterial infections remain common and alternative therapeutic solutions are urgently needed^30^. Despite the growing body of literature on green-synthesized metal oxide nanoparticles, few studies have integrated fungal metabolite profiling, CuO/ZnO nanocomposite biosynthesis, and molecular evaluation of anti-virulence activity against clinically isolated multidrug-resistant (MDR) *E. coli*. To the best of our knowledge, this is the first study to: (i) employ a molecularly identified *A. terreus* isolate for the eco-friendly synthesis of a CuO/ZnO nanocomposite; (ii) comprehensively characterize the fungal bioactive metabolites using both HPLC and GC–MS to correlate metabolite composition with nanoparticle formation; (iii) evaluate the antibacterial efficacy of the biosynthesized CuO/ZnO nanocomposite against a clinically confirmed MDR *E. coli* strain isolated from wound infections and deposited in GenBank; and (iv) investigate, at the molecular level, the impact of the nanocomposite on key virulence and quorum-sensing genes (*fimH*, *papC*, *toxA*, and *luxS*). This integrative approach, combining green nanotechnology, phytochemical profiling, and gene expression analysis, provides mechanistic insight into the anti-virulence potential of CuO/ZnO nanocomposites beyond conventional bactericidal assessment. The overall design of this study follows a translational logic: soil-derived fungi were isolated as sustainable biological factories capable of secreting reductive metabolites for green nanocomposite synthesis, while clinically isolated MDR *E. coli* strains were selected as relevant pathogenic targets to evaluate the biomedical applicability of the synthesized material. The integration of environmental fungal biotechnology with clinically relevant bacterial isolates enables the development and direct validation of eco-friendly nanomaterials against real-world multidrug-resistant pathogens. Thus, the sequential isolation of fungal and bacterial strains forms a coherent framework linking green synthesis to therapeutic application. The use of a clinically isolated MDR *E. coli* strain rather than a standard laboratory reference strain was intentional to ensure clinical relevance. Hospital-derived MDR isolates possess complex resistance mechanisms and virulence determinants that better represent real-world therapeutic challenges. While antibiotic-sensitive strains are generally easier to inhibit and are commonly used for preliminary screening, demonstrating efficacy against a resistant strain provides stronger evidence of antimicrobial potential. Therefore, this study prioritized evaluation against an MDR isolate as a stringent model to assess the effectiveness of the biosynthesized CuO/ZnO nanocomposite under clinically relevant conditions.

## Materials and methods

### Collecting soil specimens for fungus isolation and purification

To obtain a fungal isolate capable of mediating the green synthesis of CuO/ZnO nanocomposites, soil samples were collected from agricultural fields in Zagazig, Sharkia, Egypt. In April 2024, a 3.0 g soil sample was taken in a tiny, sterile sealed bag from various depths beneath the surface of the soil (from just under the surface down to one foot) in Zagazig, Sharkia, Egypt (Latitude: 30° 33’ 59.99” N, Longitude: 31° 29’ 59.99” E). To reduce the number of microbes other than fungi, the soil sample was dried in a hot-air oven at 55–60 °C. A 2.0 g soil sample was placed in 50 mL of distilled water and shaken for 35 min at 130 rpm in a orbital shaker. After serial dilution to a level of 10^− 10^, the solutions were kept at 27 ± 2 °C. Next, 100 µL aliquots of the appropriate dilution were spread onto starch acetate agar plates (pH 6.7) and incubated for 7 days. Fungal colonies were selected based on their morphology and then subcultured onto malt extract agar plates. The isolates were stored at 4 °C, and the culture filtrate was collected for further analysis^31^. Genomic DNA was extracted using the cetyltrimethylammonium bromide (CTAB) method. The internal transcribed spacer (ITS) region of the fungal rDNA was amplified using the primers ITS1f (5’-CTTGGTCAATTAGACGAAGTAA-3’) and ITS4 (5’-TCCTCCCCGTATTGATATGC-3’). The PCR products were sequenced using the BigDye Terminator method on an ABI Prism DNA sequencer. The obtained sequence was analyzed using the BLAST algorithm at the National Center for Biotechnology Information (NCBI) to identify closely related sequences. A phylogenetic tree was constructed using the neighbor-joining method in MEGA 5.0^32^. The obtained *A. terreus* isolate was selected for nanocomposite synthesis based on its rapid growth rate, high extracellular metabolite production capacity, and previously reported efficiency in fungal-mediated nanoparticle biosynthesis. Its ability to secrete high levels of bioactive compounds into the culture filtrate was considered advantageous, as these compounds can act as both reducing and capping agents during CuO/ZnO nanocomposite formation.

### Bacterial isolation

To isolate a clinically relevant multidrug-resistant (MDR) *Escherichia coli* strain for subsequent antibacterial testing, wound swabs were collected from patients in Iraqi hospitals. Wound swabs were collected from male patients attending the surgery departments at General Heet and El-Ramdy Educational Hospitals in Iraq between January and June 2024. A total of 35 individuals were enrolled: 15 patients with wound infections and 20 healthy controls. Wound swabs were cultured to isolate multidrug-resistant (MDR) bacteria. Inclusion criteria were: age between 25 and 50 years, no antibiotic use in the preceding two weeks, and absence of chronic diseases. The control group consisted of 20 healthy males who met the same inclusion criteria. This study was approved by the Research Ethics Committee of [Faculty of Science at Al-Azhar University], Egypt (Approval No. FSC010102024). All methods were performed in accordance with relevant national and international guidelines and regulations, including the Declaration of Helsinki and the Arabian Code of Ethics for Scientific Research. Written informed consent was obtained from all participants prior to sample collection. Patient confidentiality and data privacy were strictly maintained throughout the study.

Bacteria were isolated and identified from the collected specimens using the standard techniques^33^. The specimens were first inoculated into nutrient broth and incubated, then developing colonies were subcultured onto nutrient agar plates^34^. Subcultures were performed on selective media including MacConkey agar (Sigma, Egypt) and blood agar. Pure bacterial strains were obtained by repeated subculturing on nutrient agar plates incubated at 38 °C for 24 h^35^. Gram staining and the KOH test were used to examine the morphological and Gram reaction characteristics of the isolates. Biochemical tests included urease, oxidase, and catalase assays^33,34^. Although several bacterial isolates were obtained from clinical wound samples, this study focused on the confirmed MDR *E. coli* isolate for subsequent molecular characterization and antibacterial assessment, as resistant strains represent a greater therapeutic challenge than antibiotic-sensitive isolates.

### Screening for sensitivity

To confirm the multidrug-resistant phenotype of the isolated *E. coli*, antibiotic susceptibility testing was performed using the disc diffusion method according to CLSI guidelines. Antibiotic susceptibility testing was performed using the disc diffusion method on Mueller-Hinton agar according to CLSI guidelines^36^. After cooling to 42 °C, the medium was poured into Petri plates to a uniform depth of approximately 4 mm. The plates were then kept at 36 °C for 10–20 min to dry the surface. A sterile cotton swab was dipped into the bacterial suspension and used to inoculate the plates. The swab was streaked evenly over the entire agar surface, and the plate was rotated approximately 60 degrees between streaks to ensure uniform distribution. The dish was inoculated and then allowed to air dry for a few minutes at the ambient temperature. Antibiotic discs were placed on the agar surface using sterile forceps and gently pressed to ensure contact. Plates were inverted and incubated within 20 min of disc application to prevent moisture accumulation^37^. Ten antibiotic discs (Oxoid, UK) were placed on the inoculated plates using sterile forceps and gently pressed down to ensure firm contact with the agar. After incubation at 37 °C for 18–24 h, the diameters of the inhibition zones were measured in millimeters and interpreted according to CLSI breakpoints^36^.

### Phylogenetic analysis and 16 S RNA analysis

To molecularly identify the bacterial isolate and confirm its species, 16 S rRNA gene sequencing and phylogenetic analysis were conducted. Bacterial isolates were grown overnight in Luria-Bertani broth. Cells were harvested by centrifugation at 12,000 × g for 5 min and washed three times with 0.85% NaCl. Genomic DNA was extracted using the JET Genomic DNA Purification Kit (Thermo Scientific, USA) according to the manufacturer’s protocol. The 16 S rRNA gene was amplified using the universal primers: forward primer 5′-GGTCAGTCGATTGCTAACCG-3′ and reverse primer 5′ TTGGACACTGCGGTCGCATAGTC-3′. PCR amplification was performed in a total reaction volume of 25 µL containing 12.5 µL of 2× PCR Master Mix (Thermo Scientific, USA), 1 µL of each primer (10 pmol), 2 µL of template DNA, and nuclease-free water. Raw sequencing data were edited using Finch TV version 1.4.2. The strain’s 16 S rRNA sequences were examined using the National Centre for Genetic Information’s (NCBI) BLAST (N) tool (MD, USA). Multiple sequence alignment was performed using ClustalW 2.2. A phylogenetic tree was constructed using the neighbor-joining method in MEGA X^38^.

### Detection of bioactive compounds in the fungal filtrate

To identify the reducing and stabilizing metabolites present in the *A. terreus* culture filtrate responsible for mediating nanoparticle synthesis, HPLC and GC-MS analyses were performed. The fungus was cultured in yeast extract broth for 7 days, and the mycelium was removed by filtration through Whatman filter paper. The filtrate was centrifuged at 10,000 rpm for 10 min, and the supernatant was extracted with methanol (1:1, v/v) to concentrate the metabolites^39^.

For GC-MS: the bioactive compounds in the fungal filtrate were identified using gas chromatography-mass spectrometry. GC-MS analysis was performed using an Agilent 5710 automated sampler, a flame ionization detector (FID), and an Rt-540 column (100.0 m × 0.94 mm × 0.23 μm; Agilent, USA). Data were acquired using X-calibur software (Shimadzu, Japan). Helium was used as the carrier gas at a split ratio of 100:1. The pre-run was 10 min, and the equilibrium time was 0.8 min. The temperature program consisted of two ramps: first, from 49 °C to 209 °C at 8 °C/min, and then to 288 °C at 6 °C/min. The final temperature was 288 °C, and the carrier gas flow rate was 1.6 mL/min. Compounds were identified by comparing their mass spectra with those in the NIST 14 and 14 s libraries. Calibration curves were constructed using internal standards^39^.

For HPLC: The polyphenolic content of fungal filtrate extract was analyzed using a Waters HPLC system (USA) with two pumps and a UV/Vis detector. Phenolic acids were separated on a C18 column (123.0 mm × 4.60 mm, 5.2 μm particle size) using a mobile phase consisting of 49.7% (v/v) phosphoric acid and 0.13% methanol. The flow rate was 1.1 mL/min under isocratic conditions and detection was performed at 295 nm. Flavonoids were analyzed using an isocratic mobile phase of methanol: water (50:50, v/v) adjusted to pH 2.6 with phosphoric acid^40^.

### Biosynthesis and characterization of CuO/ZnO nanocomposite

To synthesize the CuO/ZnO nanocomposite using fungal metabolites and to characterize its physicochemical properties, the following procedures were carried out. The fungus filtrate was mixed with aqueous solutions of copper and zinc salts (copper acetate and zinc acetate hexahydrate) and continuously agitated for 30 min. The phytochemicals in the filtrate act as reducing agents, converting Cu²⁺ and Zn²⁺ ions into metal oxide precursors, and as capping agents to prevent particle agglomeration. The mycosynthesis mechanism is primarily mediated by extracellular biomolecules secreted by *A. terreus* into the culture filtrate. These biomolecules include phenolic acids, flavonoids, proteins, enzymes, and other reductive metabolites identified by HPLC and GC–MS analysis. During synthesis, these functional groups (–OH, –COOH, –NH₂) interact electrostatically with Cu²⁺ and Zn²⁺ ions. The phenolic and flavonoid compounds donate electrons, facilitating the bioreduction of metal ions into their respective oxide precursors. Simultaneously, proteins and polysaccharides act as capping and stabilizing agents, preventing uncontrolled aggregation by forming a protective organic layer around the growing nuclei. To remove any residual impurities or excess fungal biomass components, the reaction mixture was filtered and subsequently dried prior to calcination^41^.

The process proceeds through sequential stages: (i) activation phase, where metal ions bind to negatively charged functional groups on fungal metabolites; (ii) nucleation phase, involving reduction and formation of CuO and ZnO nuclei; (iii) growth phase, where controlled particle enlargement occurs via metabolite-mediated stabilization; and (iv) calcination phase (450 °C), during which organic residues decompose, resulting in crystalline CuO/ZnO nanocomposite formation. This biomass-mediated synthesis enables simultaneous reduction and stabilization without requiring external chemical reducing agents^41^. The green synthesis of metal oxide nanocomposites is strongly influenced by several physicochemical parameters, including pH, temperature, precursor concentration, and reaction time. In the present study, the reaction conditions were carefully controlled to ensure efficient reduction of Cu²⁺ and Zn²⁺ ions and uniform nanocomposite formation. The pH of the reaction mixture plays a crucial role in modulating the ionization state of fungal metabolites and metal ion solubility, thereby affecting nucleation and particle growth. Reaction temperature influences reduction kinetics and crystallinity of the formed nanoparticles, while precursor salt concentration determines nucleation density and particle size distribution. Additionally, sufficient reaction time is necessary to allow complete bioreduction and stabilization of nanoparticles by fungal metabolites. These parameters collectively contribute to controlling particle morphology, crystallite size, and stability of the CuO/ZnO nanocomposite^25,41^. The present study focused on reproducible green synthesis under controlled reaction conditions. Although reaction parameters were carefully maintained to ensure consistent nanocomposite formation, a systematic batch-mode optimization study evaluating yield variation under different reaction volumes, precursor ratios, or scaling approaches was not performed. The synthesis was conducted under a fixed batch configuration to maintain uniformity and allow accurate physicochemical and biological evaluation.

The biosynthesized CuO/ZnO nanocomposite was characterized using various analytical techniques to determine its physical, optical, and morphological properties. The crystalline structure of the biosynthesized CuO/ZnO nanocomposite was analyzed using X-ray diffraction (XRD) with Cu Kα radiation (λ = 0.15406 nm) over a 2θ range of 20°–80°, following standard powder diffraction procedures for phase identification. Diffraction peaks were indexed using Joint Committee on Powder Diffraction Standards (JCPDS) database files to confirm crystalline phases. The average crystallite size was calculated using the Debye–Scherrer equation, a widely accepted method for estimating nanoscale crystallite dimensions from peak broadening. Surface functional groups involved in reduction and stabilization were examined using Fourier Transform Infrared Spectroscopy (FTIR) in the range of 4000–400 cm⁻¹, following standard vibrational spectroscopy protocols for nanoparticle characterization. The morphology and particle size distribution were investigated using Transmission Electron Microscopy (TEM), operating at an accelerating voltage of 200 kV, allowing direct visualization of nanoscale structures. Particle size measurements were performed using ImageJ software from multiple micrographs to ensure statistical reliability. The optical properties of the nanocomposite were evaluated using UV–Visible spectroscopy within the range of 200–800 nm to determine characteristic absorption peaks and estimate the optical band gap using Tauc plot analysis, as described in standard semiconductor nanoparticle characterization methods. All characterization techniques were performed according to established nanomaterial analysis protocols widely reported in metal oxide nanoparticle research^23–26^. The physicochemical characteristics observed in the present study can be partially attributed to the controlled synthesis parameters. It is well established that alkaline pH conditions generally enhance nanoparticle formation by promoting deprotonation of functional groups in fungal metabolites, thereby increasing their reducing and capping efficiency. Similarly, elevated calcination temperature (450 °C in this study) contributes to improved crystallinity and phase purity of CuO and ZnO components. Precursor concentration directly affects supersaturation levels, which govern nucleation rate and final particle size; excessive concentrations may lead to aggregation, whereas optimized ratios favor homogeneous nanocomposite formation. Reaction time also influences the completeness of metal ion reduction and stabilization processes. The controlled optimization of these factors likely contributed to the nanoscale size (~ 45 nm), high crystallinity, and enhanced antibacterial performance observed for the CuO/ZnO nanocomposite as illustrated in other reports^24,29^.

### Screening for antibacterial activity against MDR pathogenic bacteria

To evaluate the antibacterial efficacy of the biosynthesized CuO/ZnO nanocomposite against the clinically isolated MDR *E. coli* strain, disc diffusion, minimum inhibitory concentration (MIC), and time-kill assays were performed. Mueller-Hinton agar plates were streaked with a suspension of the MDR *E. coli* isolate. Wells (1.0 cm diameter) were punched in the agar, and 100 µL of the CuO/ZnO nanocomposite suspension was added to each well. The plates were incubated at 37 °C for 24 h, after which the diameters of the inhibition zones were measured^33^. The minimum inhibitory concentration (MIC) of the biosynthesized CuO/ZnO nanocomposite was determined using the standard broth microdilution method according to CLSI guidelines. Briefly, two-fold serial dilutions of the nanocomposite were prepared in Mueller–Hinton broth in 96-well microtiter plates to obtain concentrations ranging from 3.12 to 500 µg/mL. A standardized bacterial suspension equivalent to 0.5 McFarland standard (~ 1 × 10⁸ CFU/mL) was diluted and inoculated into each well to achieve a final concentration of approximately 1 × 10⁶ CFU/mL. Plates were incubated at 37 °C for 18–24 h. The MIC was defined as the lowest concentration of the nanocomposite that showed no visible bacterial growth compared to the untreated control^36^. Time-kill kinetics were performed to evaluate the bactericidal activity of the CuO/ZnO nanocomposite over time. The MDR *E. coli* culture was exposed to the nanocomposite at concentrations equivalent to 1× MIC and 2× MIC. At predetermined time intervals (0, 2, 4, 6, 8, and 24 h), aliquots were withdrawn, serially diluted in sterile saline, and plated onto Mueller–Hinton agar plates. After incubation at 37 °C for 24 h, colony-forming units (CFU/mL) were counted. Bactericidal activity was defined as a ≥ 3 log₁₀ reduction in CFU/mL compared to the initial inoculum^36^.

### Gene expression testing

To assess the effect of the CuO/ZnO nanocomposite on the expression of virulence and quorum-sensing genes in MDR *E. coli*, quantitative reverse transcription PCR (qRT-PCR) was performed. The MDR *E. coli* isolate was treated with a sub-inhibitory concentration (½ MIC) of the CuO/ZnO nanocomposite; untreated cultures served as controls. Bacterial cells were taken after 18–24 h of incubation at 37 °C, and total RNA was extracted using the GeneJET RNA Purification Kit (Thermo Scientific, USA) following the manufacturer’s instructions. DNase I treatment was performed using the DNase I, RNase-free kit (Thermo Scientific, USA) to eliminate genomic DNA contamination. First-strand cDNA synthesis was carried out using the RevertAid First Strand complementary DNA (cDNA) Synthesis Kit (Thermo Scientific, USA). Quantitative real-time PCR was conducted using SYBR Green PCR Master Mix (Applied Biosystems, USA) in a StepOnePlus™ Real-Time PCR System (Applied Biosystems, USA). The specific primers used for *fimH*, *papC*,* toxA*,* luxS*, and 16 S rRNA genes are listed in (Supplementary Table 1). The quantitative real-time PCR (qRT-PCR) conditions included an initial denaturation at 95 °C for 10 min, followed by 40 cycles of 95 °C for 15 s and 60 °C for 30 s. To quantify relative gene expression, treated samples were compared to untreated controls using the 2⁻ΔΔCt technique^35,36^.

### Statistics assessment

To determine the statistical significance of the experimental data, all results were analyzed using appropriate parametric tests. All experiments were performed in triplicate (*n* = 3), and the results are presented as mean ± standard deviation (SD). Statistical analyses were conducted using GraphPad Prism version 9 (GraphPad Software, USA). Differences between treated and control groups were analyzed using one-way ANOVA followed by Tukey’s post hoc test. A *p*-value < 0.05 was considered statistically significant.

## Results and discussion

The growing incidence of MDR bacterial infections, notably *Escherichia coli*, poses a serious threat to public health and reduces the efficacy of traditional antibiotics. This problem has sparked increased research in alternate antimicrobial methods that not only decrease bacterial growth but also reduce virulence and pathogenicity. In this context, biologically manufactured metal oxide nanocomposites have emerged as attractive possibilities due to their increased antibacterial potency, lower toxicity, and environmentally friendly manufacturing^42,43^. The current study uses microbial isolation, molecular identification, green nanotechnology, and molecular biology techniques to examine the antibacterial and anti-virulence properties of a biosynthesized CuO/ZnO nanocomposite against MDR *E. coli.*

### The obtained bacterium isolation and identification

**Fig. 1:**
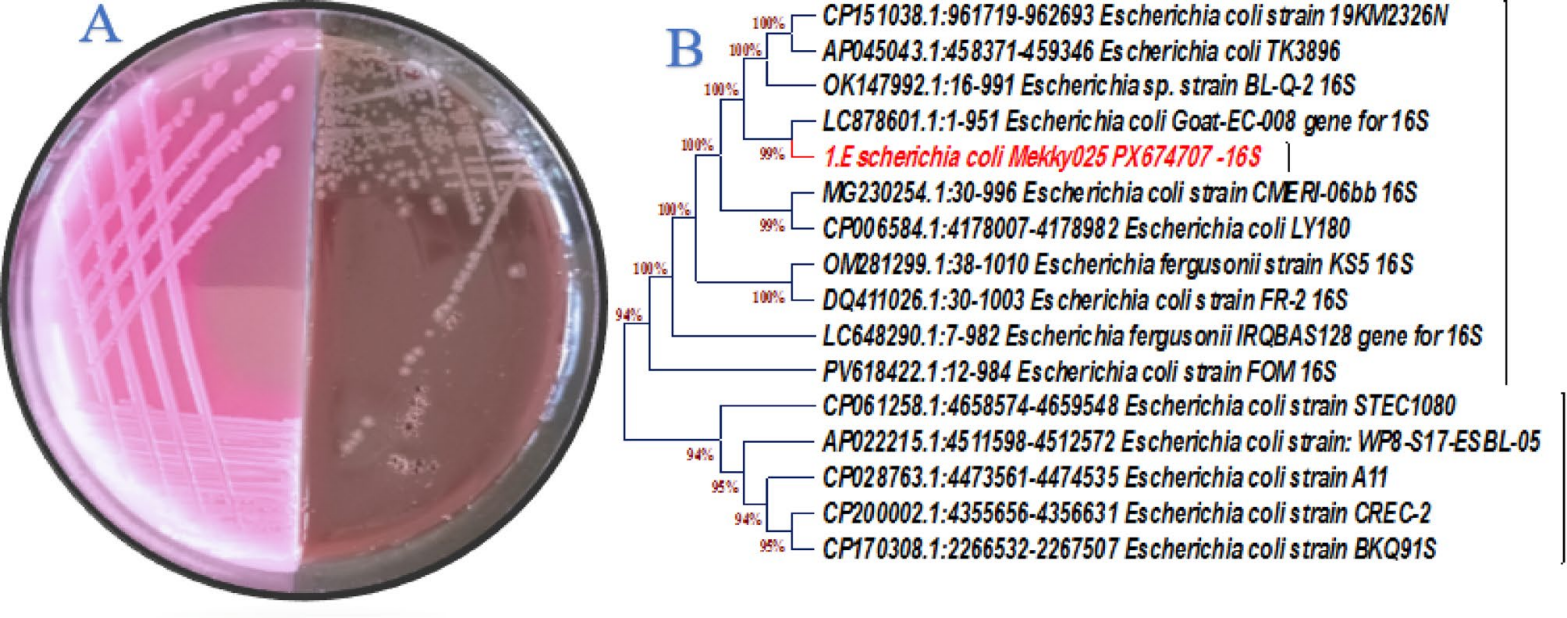
(**A**) Isolated bacteria cultured on MacConkey agar and blood agar media; (**B**) Phylogenetic analysis of the MDR *E. Coli* bacteria that was isolated from the wound.

**Fig. 2:**
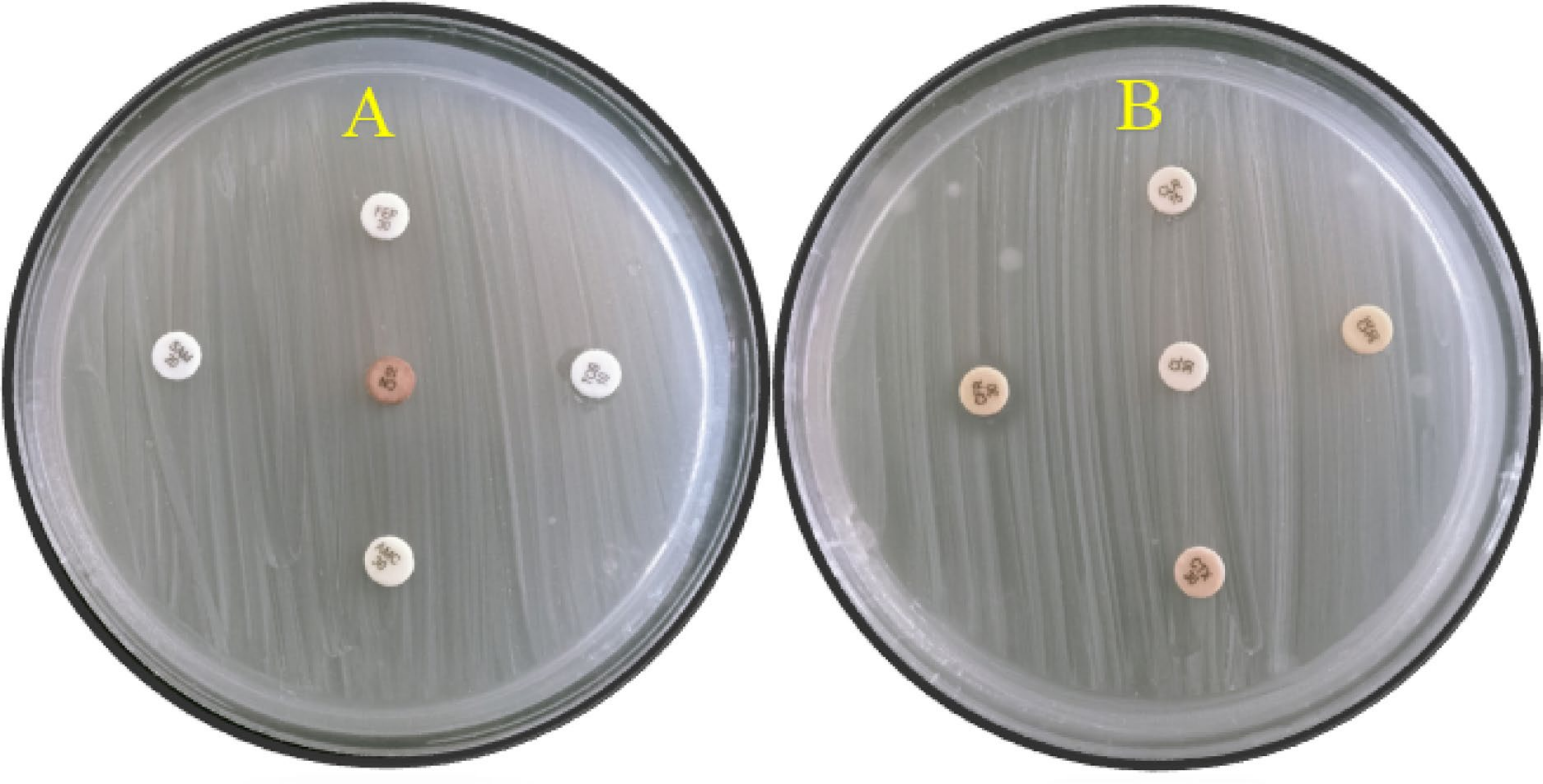
Antibiotic sensitivity compared to isolated bacteria from wounds (the antibiotic discs used: (**A**) E stands for erythromycin, DA for clindamycin, NOR for norfloxacin, AM for amoxicillin, and AMC for amoxicillin-clavulanic acid. (**B**) OX: oxacillin, FOX: cefoxitin, TN: ciprofloxacin, LZD: linezolid, MFX: moxifloxacin).

The isolated bacterium formed wet, grayish-white colonies on blood agar and exhibited non-hemolytic (gamma-hemolysis) activity. On MacConkey agar, the colonies appeared pink due to lactose fermentation, which lowers the pH and causes the neutral red indicator to change color in the presence of bile salts (Fig. 1A). The drug-resistant bacterial strain was identified as *E. coli* using 16 S rRNA, and it was deposited in the gene bank with code PX674707 (https://www.ncbi.nlm.nih.gov/nuccore/PX674707). Phylogenetic analysis showed 99.7% sequence similarity with existing GenBank isolates (Fig. 1B). Antibiotic susceptibility testing revealed that the Gram-negative isolate was resistant to all antibiotics tested. The isolate was confirmed as *Escherichia coli* based on morphological, biochemical, and molecular characterization (Fig. 2). The isolation of an MDR *E. coli* strain resistant to all tested antibiotics is consistent with previous reports highlighting the alarming increase in extensively drug-resistant organisms in clinical and environmental settings^44,45^. Previous studies have shown that such resistance complicates therapy and underscores the need for alternative antimicrobial strategies, including nanotechnology-based interventions^23,46^.

### Molecular identification of soil fungus used for CuO/ZnO nanocomposite biosynthesis

**Fig. 3:**
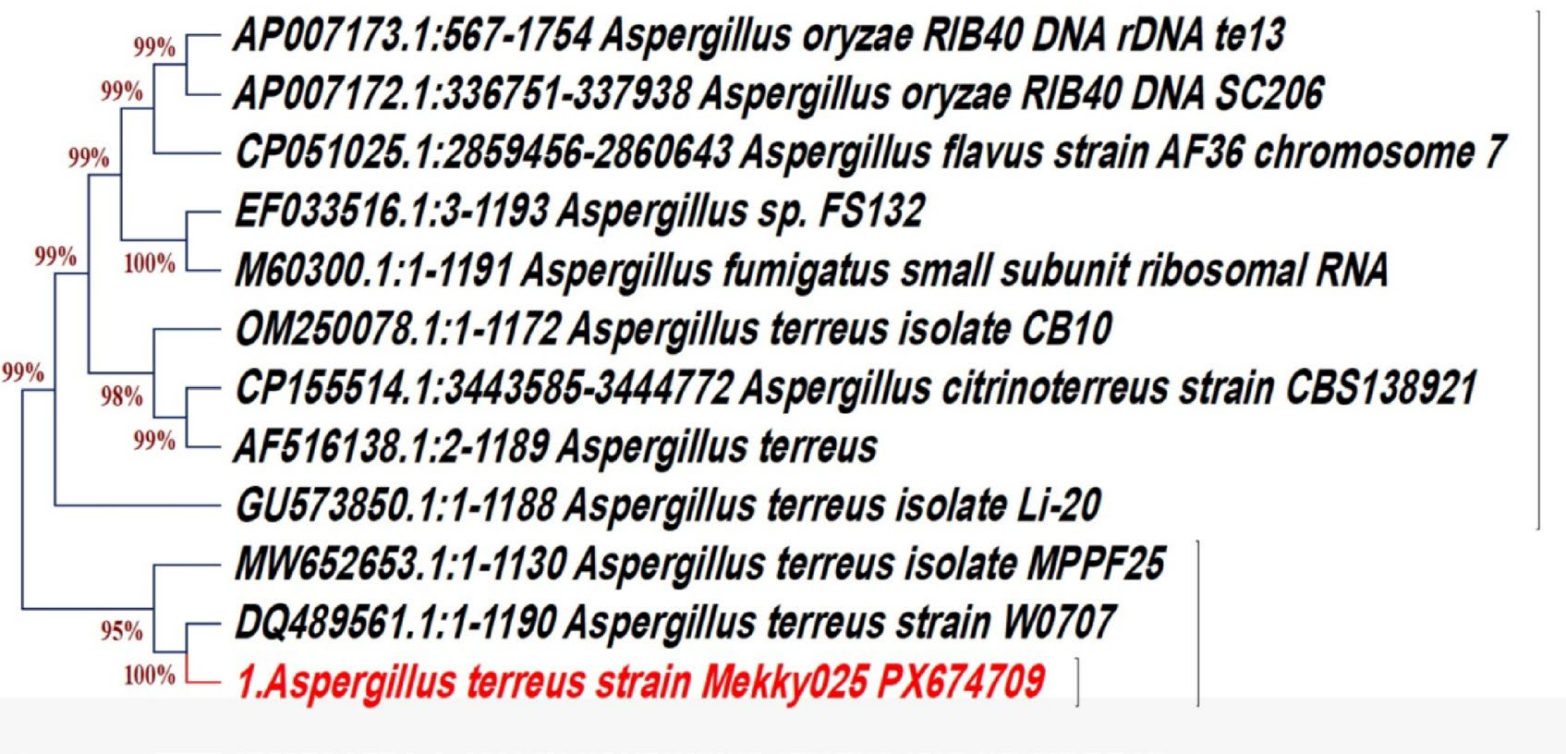
Phylogenetic tree for *A. terreus* that produced the CuO/ZnO nanocomposite.

The soil-derived fungus was isolated and molecularly identified for its potential use in the green biosynthesis of the CuO/ZnO nanocomposite, based on its ability to secrete extracellular metabolites capable of mediating metal ion reduction and stabilization. Phylogenetic analysis based on 18 S rRNA gene sequencing identified the soil-derived fungus as Aspergillus terreus, showing 99.6% sequence similarity with GenBank reference strains. The sequence was deposited in GenBank under accession number PX674709 (https://www.ncbi.nlm.nih.gov/nuccore/PX674709 ) (Fig. 3). The identification of A. terreus further supported its selection for nanocomposite synthesis due to its established role in producing extracellular metabolites capable of mediating metal ion reduction and nanoparticle stabilization, as reported in previous biosynthesis studies^47,48^. The high sequence similarity observed in phylogenetic analysis confirms the reliability of molecular identification methods, as previously reported in fungal-mediated nanoparticle synthesis studies^49^.

### Determination of different compounds in *A. terreus* filtrate using HPLC and GC-MS

**Fig. 4:**
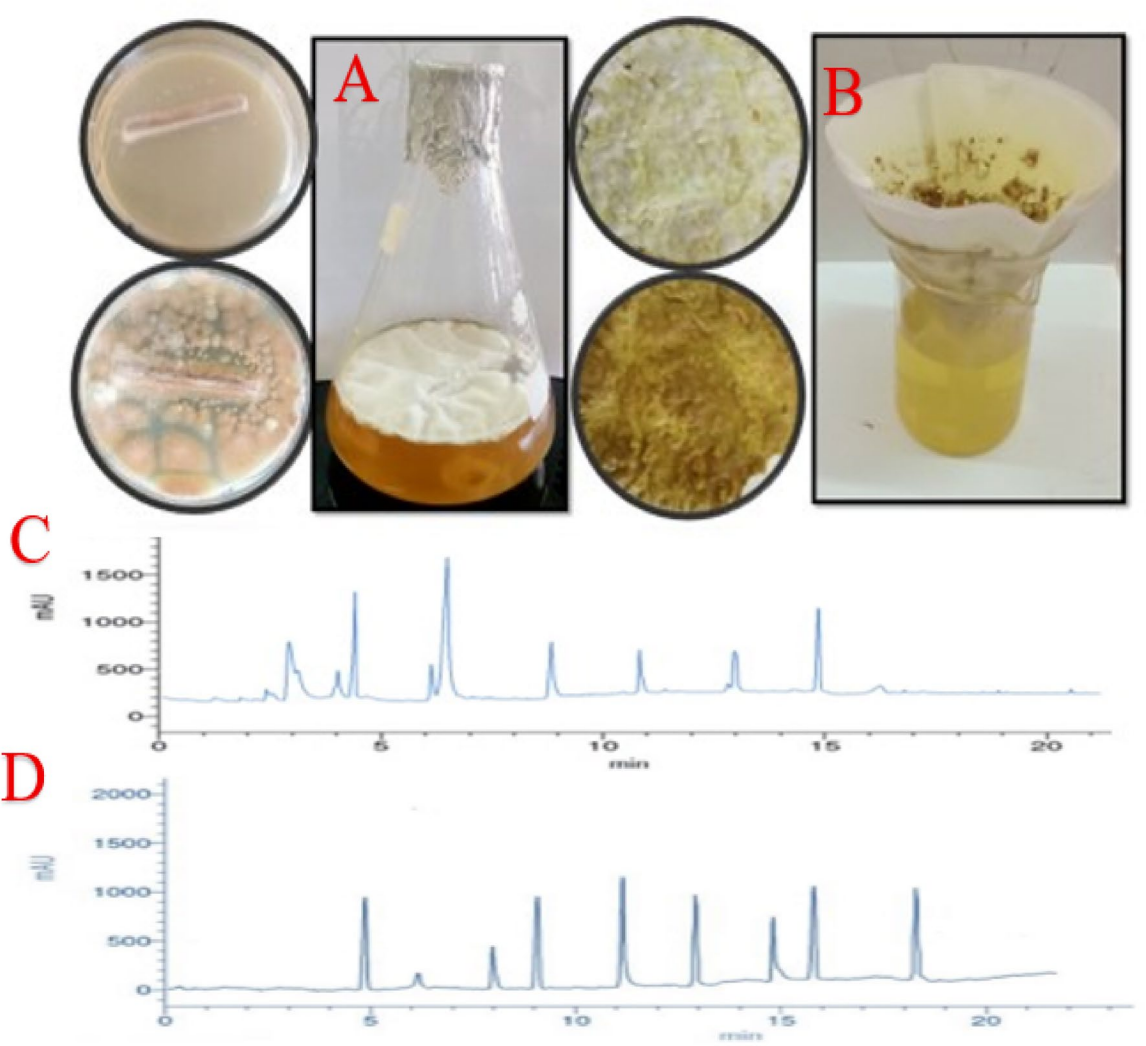
(**A**) Cultivation of *A. terreus on broth media, *(**B**) *A. terreus. *filtrate, (**C**) Phenolic compounds in *A. terreus. *filtrate via HPLC, (**D**) Flavonoids in in *A. terreus. *filtrate via HPLC.

**Fig. 5:**
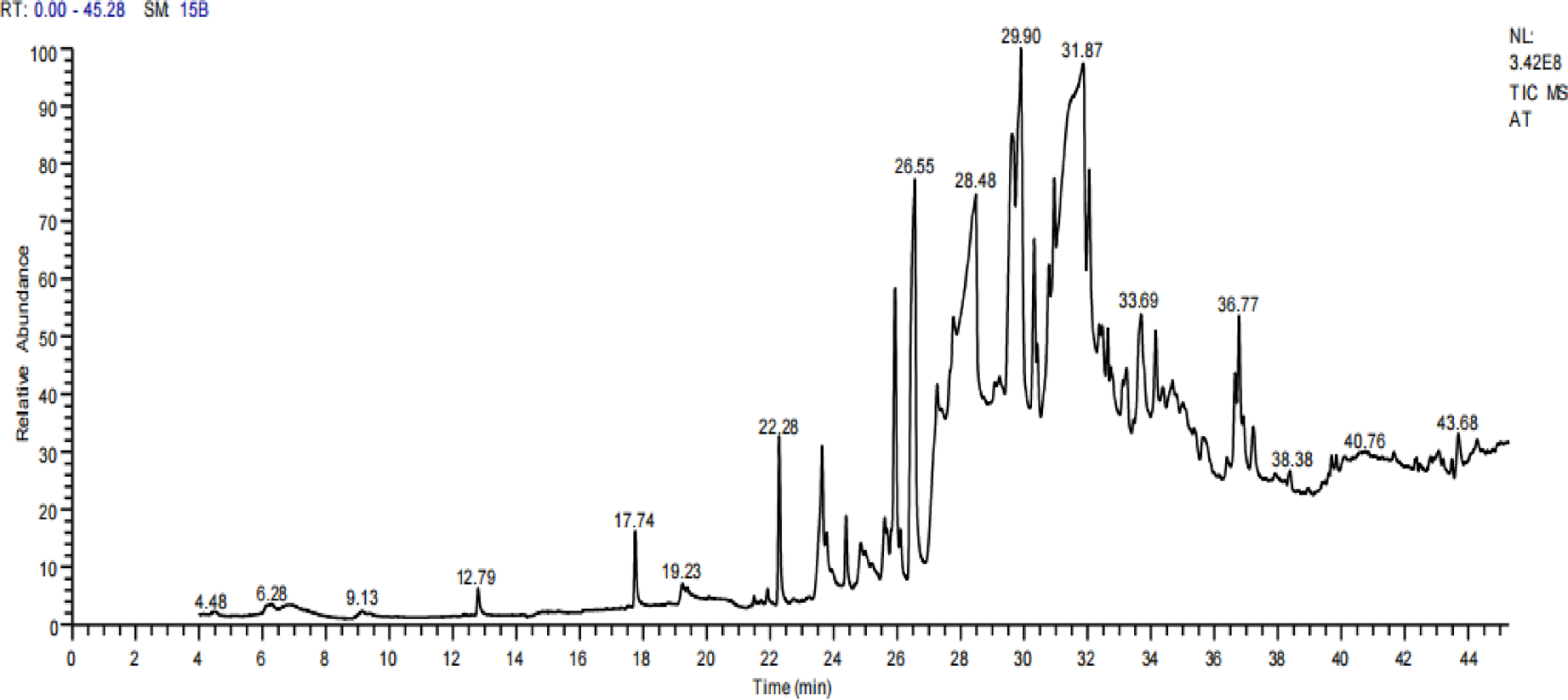
GC-MS examination of *A. terreus. *filtrate.

HPLC analysis of the *A. terreus* filtrate revealed the presence of nine phenolic acids, with ferulic acid and gallic acid being the most abundant (Table 1 and Fig. 4C). Additionally, nine flavonoids were detected, with apigenin and catechin being the predominant ones (Table 1 and Fig. 4D). Besides, twelve various molecules could be seen in the fungal filtrate which were: 2-Propenoic acid, 3-phenyl-, methyl ester; Benzaldehyde; Benzenepropanenitrile; Cinnamaldehyde, (E)-; Benzeneacetaldehyde; 2-Propenal, 3-phenyl-; 2-Propenal, 2-methyl-3-phenyl-; Ethanone, 2,2-dihydroxy-1-phenyl-; Benzenemethanol, à-methyl-à-propyl-; trans-Cinnamic acid; trans-13-Octadecenoic acid and 1,4-Ethanonaphthalene, 1,2,3,4-tetrahydro-. Besides, Cinnamaldehyde, (E)-; 9, 12-Octadecenoic acid (Z, Z)-, and Benzenemethanol, à-methyl-à-propyl- represented the major molecules in the fungal filtrate (Table 2 and Fig. 5). HPLC and GC-MS investigations confirmed the presence of phenolic acids, flavonoids, and aromatic substances with reducing and stabilizing characteristics. Previous studies have shown that compounds such as ferulic acid, gallic acid, apigenin, and cinnamaldehyde play a significant role in nanoparticle synthesis and stabilization^50,51^. Despite its chemical richness, the fungal filtrate alone showed no antibacterial activity against MDR *E. coli*, consistent with previous findings suggesting that free bioactive compounds may be insufficient to overcome robust bacterial defense mechanisms^52,53^.

**Table 1:** Different phenolic compounds and flavonoids identified using HPLC testing for *A. terreus* filtrate.

RT	Identified compound (phenolic compounds)	Conc (mg/ml)
5.3	Chlorogenic acid	14.79
6.2	Cinnamic acid	3.75
8.1	Ellagic acid	6.73
9.3	Ferulic acid	15.16
11.2	Gallic acid	20.71
13.1	Coumaric acid	12.27
15.3	Syringic acid	9.50
16.2	Vanillic acid	11.70
18.4	Hydroxycinnamic acid	14.63
RT	Identified compound (Flavonoids)	Conc (mg/ml)
3.1	Chryin	7.78
4.3	Apigenin	14.32
6.4	Catechin	17.52
8.9	kaempferol	6.15
10.7	Galangin	5.65
13.5	Luteolin	4.63
15.2	Rutin	10.52

**Table 2:** Different volatile compounds molecules detected in filtrate of *A. terreus*.

RT	Compound Name	Area %	Molecular Formula	Molecular Weight
5.36	Cinnamaldehyde, (E)-	39.01	C_9_H_8_O	132
7.40	Benzaldehyde	2.53	C_7_H_6_O	106
8.16	2-Propenal, 2-methyl-3-phenyl-	0.43	C_10_H_10_O	146
12.22	Ethanone, 2,2-dihydroxy-1-phenyl-	0.47	C_8_H_8_O_3_	152
13.72	2-Propenal, 3-phenyl-	9.12	C_9_H_8_O	132
14.73	Benzeneacetaldehyde	1.56	C_8_H_8_O	120
15.39	Benzenepropanenitrile	4.27	C_9_H_9_N	131
16.78	Benzenemethanol, à-methyl-à-propyl-	36.01	C_11_H_16_O	164
17.26	2-Propenoic acid, 3-phenyl-, methyl ester	0.81	C_10_H_10_O_2_	164
18.64	Ethanonaphthalene, 1,2,3,4-tetrahydro- 1,4	0.39	C_12_H_14_	158
21.15	trans-13-Octadecenoic acid	4.2	C_18_H_34_O_2_	282
30.05	trans-Cinnamic acid	1.20	C_9_H_8_O_2_	148

### Characterization of the produced CuO/ZnO nanocomposite

**Fig. 6:**
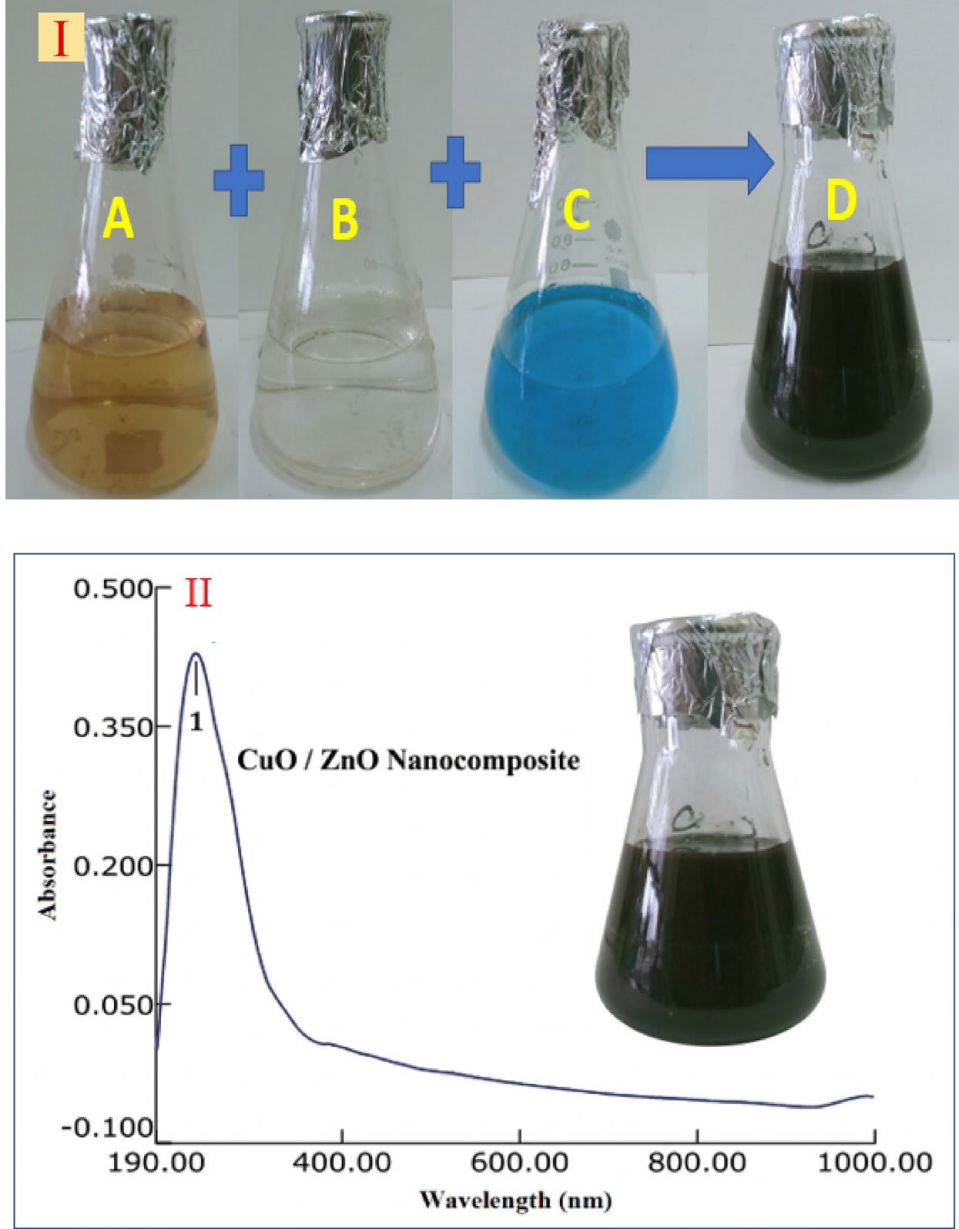
I. Biosythesis of CuO/ZnO nanocompsite using *A. terreus*. I (**A**) Fungal filterate, (**B**) Zinc acetate, (**C**) Cupper acetate solutions (**D**) The produced CuO/ZnO nanocompsite II. CuO/ZnO nanocompsite UV-spectrum,

**Fig. 7:**
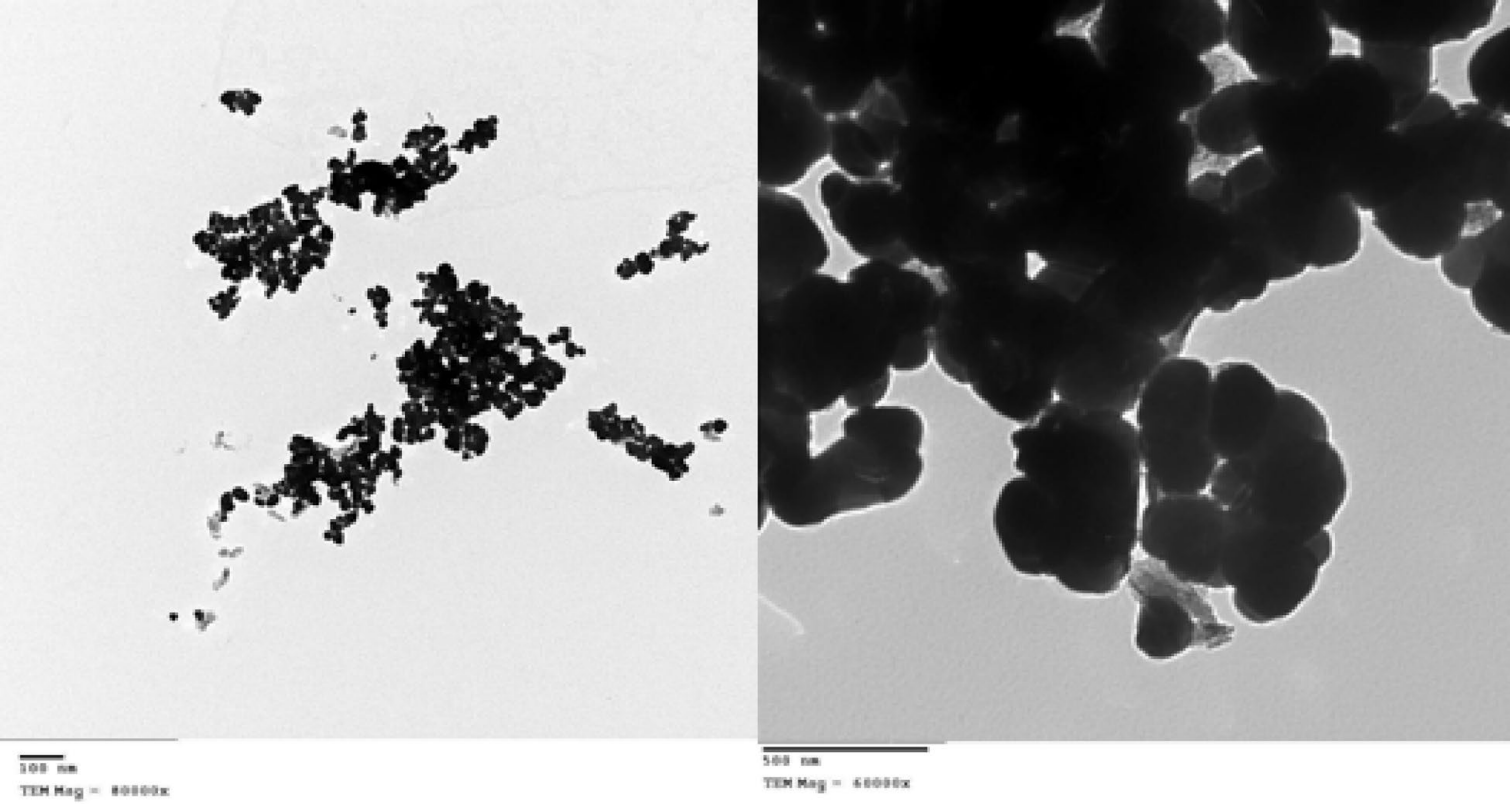
TEM for CuO/ZnO nanocompsite,.

**Fig. 8:**
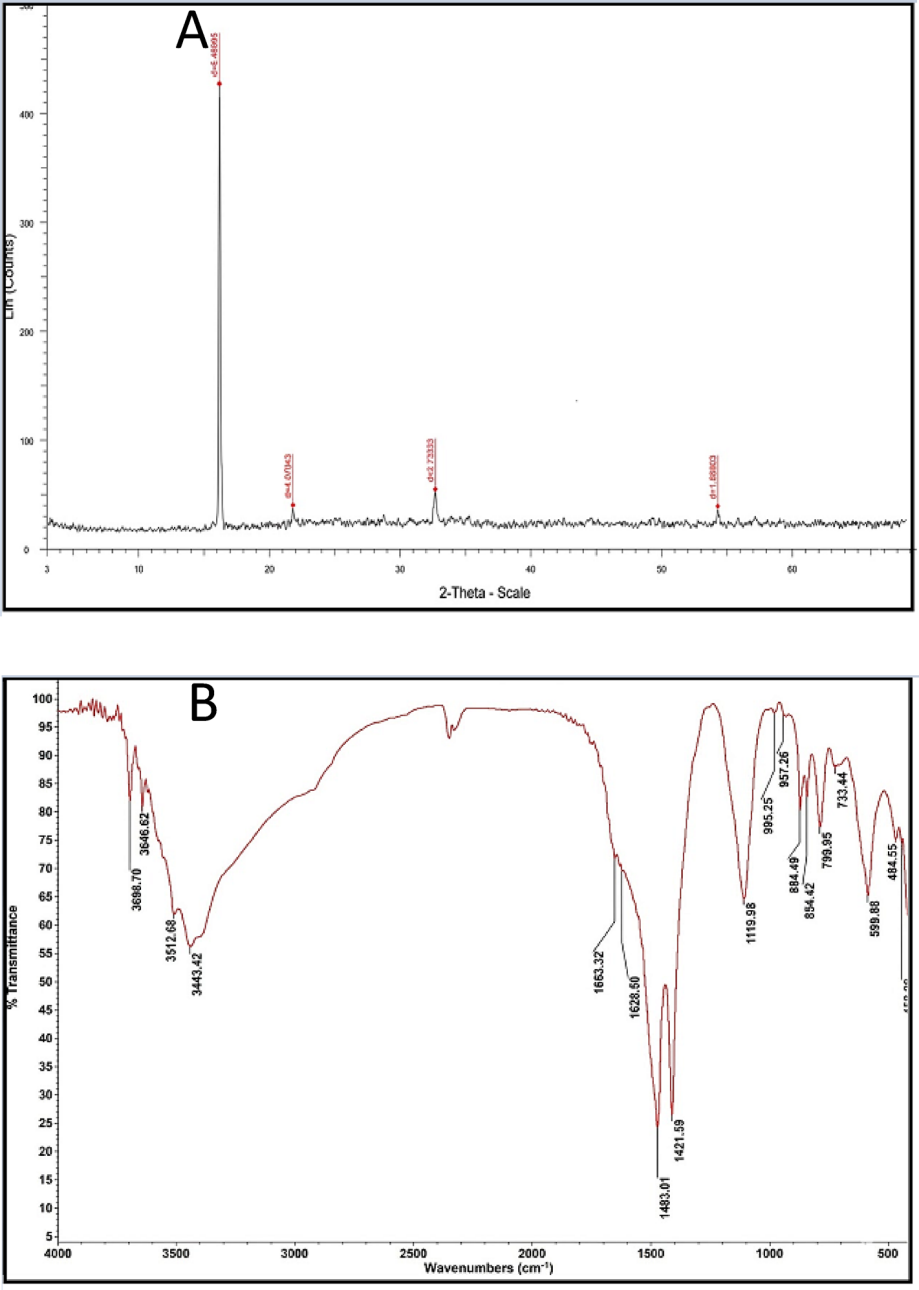
(**A**) XRD, (**B**) FTIR for prodcued CuO/ZnO nanocompsite

UV-Visible spectroscopy of the CuO/ZnO nanocomposite revealed a significant absorption band in the ultraviolet region, with a widened absorption edge at 380 nm that extends into the visible range. Compared to pure ZnO, the nanocomposite’s absorption edge shifted toward longer wavelengths. The optical band gap calculated from the Tauc plot was smaller than that of ZnO, indicating altered optical absorption behavior in the nanocomposite (Fig. 6). Transmission electron microscopy (TEM) images showed that the CuO/ZnO nanocomposite consisted of nanosized particles with predominantly spherical and slightly irregular morphologies. The average particle size was approximately 45 nm. Darker regions corresponding to CuO nanoparticles were dispersed over lighter ZnO nanoparticles, indicating the presence of both components in the composite. The particles appear to be well dispersed, with distinct borders between each nanoparticle (Fig. 7). The FTIR spectroscopy data revealed a large absorption band in the region of 3200–3600 cm⁻¹, attributable to surface-adsorbed hydroxyl groups. A faint band about 1630 cm⁻¹ was observed, indicating H-O-H bending vibrations. Metal-oxygen stretching vibrations were observed in the low wavenumber region, with bands at 500–600 cm⁻¹ assigned to Zn-O vibrations and bands in the range of 430–520 cm⁻¹ assigned to Cu-O vibrations. This confirms the presence of both oxide components (Fig. 8 A). The XRD pattern of the biosynthesized CuO/ZnO nanocomposite exhibited four characteristic diffraction peaks confirming the coexistence of both crystalline phases. The prominent peaks observed at 2θ values of approximately 34.4° and 36.2° were indexed to the (002) and (101) planes of hexagonal wurtzite ZnO (JCPDS No. 36–1451). Additionally, diffraction peaks located at approximately 35.5° and 38.7° corresponded to the (–111) and (111) crystallographic planes of monoclinic CuO (JCPDS No. 45–0937). The presence of these distinct peaks without additional impurity signals confirms the successful formation and phase purity of the CuO/ZnO heterostructured nanocomposite. The average crystallite size was estimated using the Debye–Scherrer equation, D = (Kλ)/(βcosθ), where K = 0.9, λ = 0.15406 nm (Cu Kα radiation), β is the full width at half maximum (FWHM), and θ is the Bragg angle. The calculated crystallite size was approximately 32–45 nm, indicating nanoscale crystalline formation (Fig. 8B). The CuO/ZnO nanocomposite exhibited improved optical properties, a reduced band gap, nanoscale particle size, and high crystallinity, consistent with previously reported synergistic interactions between CuO and ZnO^24,25^. The reduction in band gap is attributed to interfacial interactions between CuO and ZnO, which enhance charge separation and electron transfer across the heterojunction. This interaction reduces electron-hole recombination and increases photo-induced activity, contributing to better functional performance than the individual oxides. Furthermore, nanoscale particle size increases specific surface area, resulting in more active locations for surface reactions and communication with microbial cells^24,25^. The successful formation of the CuO/ZnO nanocomposite can be mechanistically explained by the biochemical activity of fungal biomass-derived metabolites. FTIR analysis confirmed the presence of hydroxyl, carbonyl, and amine functional groups, supporting their involvement in metal ion coordination and stabilization. The reduction of Cu²⁺ and Zn²⁺ ions is likely driven by electron-donating phenolic compounds such as gallic acid, ferulic acid, and flavonoids detected in the filtrate. These metabolites facilitate redox conversion and initiate nanoparticle nucleation^24,25^. Furthermore, extracellular proteins secreted by the fungus may bind to nanoparticle surfaces through amide linkages, controlling particle growth and morphology. During calcination, decomposition of organic capping agents results in the formation of highly crystalline CuO and ZnO phases while preserving nanoscale structure. The heterojunction formation between CuO and ZnO occurs during thermal treatment, enhancing interfacial contact and stability. Therefore, the fungal biomass plays a dual mechanistic role: (i) biochemical reduction and stabilization in the aqueous phase and (ii) structural templating that guides nanocomposite architecture after thermal conversion^24^. Several studies have reported that combining these oxides enhances ROS production, membrane disruption, and metal ion release, thereby increasing antibacterial efficacy^25,26^. Furthermore, the nanocomposite disrupts bacterial cell membranes through direct physical contact and electrostatic interactions, leading to loss of membrane integrity and leakage of cellular contents. The simultaneous release of Cu²⁺ and Zn²⁺ ions further enhance antibacterial activity by disrupting enzymatic processes and metabolic pathways in bacterial cells. Together, these synergistic effects explain the CuO/ZnO nanocomposite’s enhanced antibacterial efficacy and highlight its potential for use in antimicrobial coatings^25,26^. While the biosynthesis approach demonstrated stable nanocomposite formation and reproducible antibacterial performance, further investigation into batch-mode optimization strategies (including scaling volume, precursor concentration ratios, and reaction kinetics) would be valuable for maximizing production yield and enabling potential industrial translation. Future studies may focus on systematic yield optimization and process scalability assessment.

**Fig. 9:**
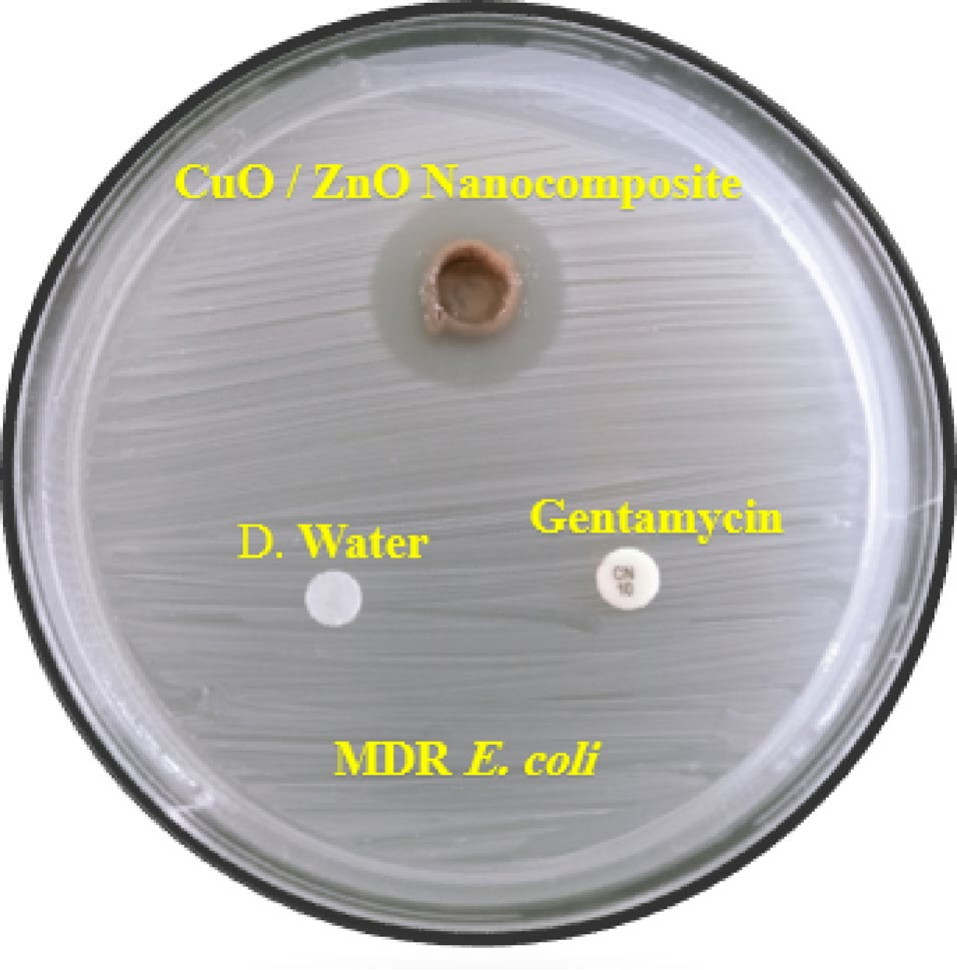
Antimicrobail impact of produced CuO/ZnO nanocompsite Compared with distilled water as a negative control and gentamycin as a positive control versus MDR *E. coli *using agar diffusion testing.

### Antibacterial action of CuO/ZnO nanocomposite against MDR *E. coli* gene expression outcome

The *A. terreus* filtrate alone showed no antibacterial activity against MDR *E. coli*. In contrast, the biosynthesized CuO/ZnO nanocomposite had a notable antibacterial impact with inhibition zone = 2.3 ± 0.4 mm and MIC = 62.5 ± 0.2 µg/mL as seen in (Fig. 9). The time-kill kinetics demonstrated a concentration- and time-dependent bactericidal effect of the CuO/ZnO nanocomposite against the MDR *E. coli* isolate. At 1× MIC, the bacterial count decreased from an initial inoculum of 6.2 log₁₀ CFU/mL to 4.8 log₁₀ CFU/mL after 4 h and further declined to 3.5 log₁₀ CFU/mL at 8 h, reaching 2.9 log₁₀ CFU/mL after 24 h. In contrast, treatment with 2× MIC resulted in a more pronounced reduction, with bacterial counts decreasing to 4.1 log₁₀ CFU/mL at 2 h, 2.7 log₁₀ CFU/mL at 4 h, and 1.8 log₁₀ CFU/mL at 8 h. A ≥ 3 log₁₀ reduction in CFU/mL compared to the initial inoculum was observed within 6–8 h at 2× MIC, confirming bactericidal activity of the nanocomposite. The untreated control showed sustained bacterial growth, reaching 8.4 log₁₀ CFU/mL at 24 h (Supplementary Table 2). Quorum sensing regulates biofilm formation, motility, and virulence factor expression; hence, disrupting it is an important technique for reducing bacterial pathogenicity without applying selective pressure for resistance^54^. Furthermore, the considerable downregulation of quorum-sensing and virulence-associated genes supports previous research indicating that metal oxide nanoparticles can disrupt bacterial communication and adhesion pathways^54,55^. The antibacterial activity of the CuO/ZnO nanocomposite can be attributed to multiple synergistic mechanisms. A primary mechanism involves enhanced generation of reactive oxygen species (ROS), including superoxide radicals and hydroxyl radicals, resulting from the heterojunction interaction between CuO and ZnO, which promotes efficient electron–hole separation^56^. These ROS induce oxidative stress within bacterial cells, leading to lipid peroxidation, protein oxidation, and DNA damage, ultimately causing cellular dysfunction and death^56,57^.

In addition, the nanoscale particle size facilitates direct interaction with the negatively charged outer membrane of Gram-negative *E. coli*, resulting in membrane destabilization, increased permeability, and leakage of intracellular components^23,52^. The gradual release of Cu²⁺ and Zn²⁺ ions further enhances antibacterial efficacy by interfering with essential enzymatic systems, disrupting metabolic pathways, and promoting intracellular oxidative imbalance^21,53^. Copper ions, in particular, may catalyze Fenton-like reactions that amplify ROS production and cellular toxicity^21^. Beyond direct bactericidal effects, metal oxide nanoparticles have been reported to interfere with quorum sensing and virulence regulation pathways^54–56^. In the present study, significant downregulation of *luxS*,* fimH*,* papC*, and *toxA* genes suggests that the CuO/ZnO nanocomposite exerts an additional anti-virulence effect, reducing bacterial adhesion, communication, and toxin production. Therefore, the antibacterial efficiency of the biosynthesized CuO/ZnO nanocomposite arises from combined ROS-mediated oxidative damage, membrane disruption, metal ion toxicity, and molecular suppression of virulence pathways^23,52–56^.

### Gene expression assay

**Fig. 10:**
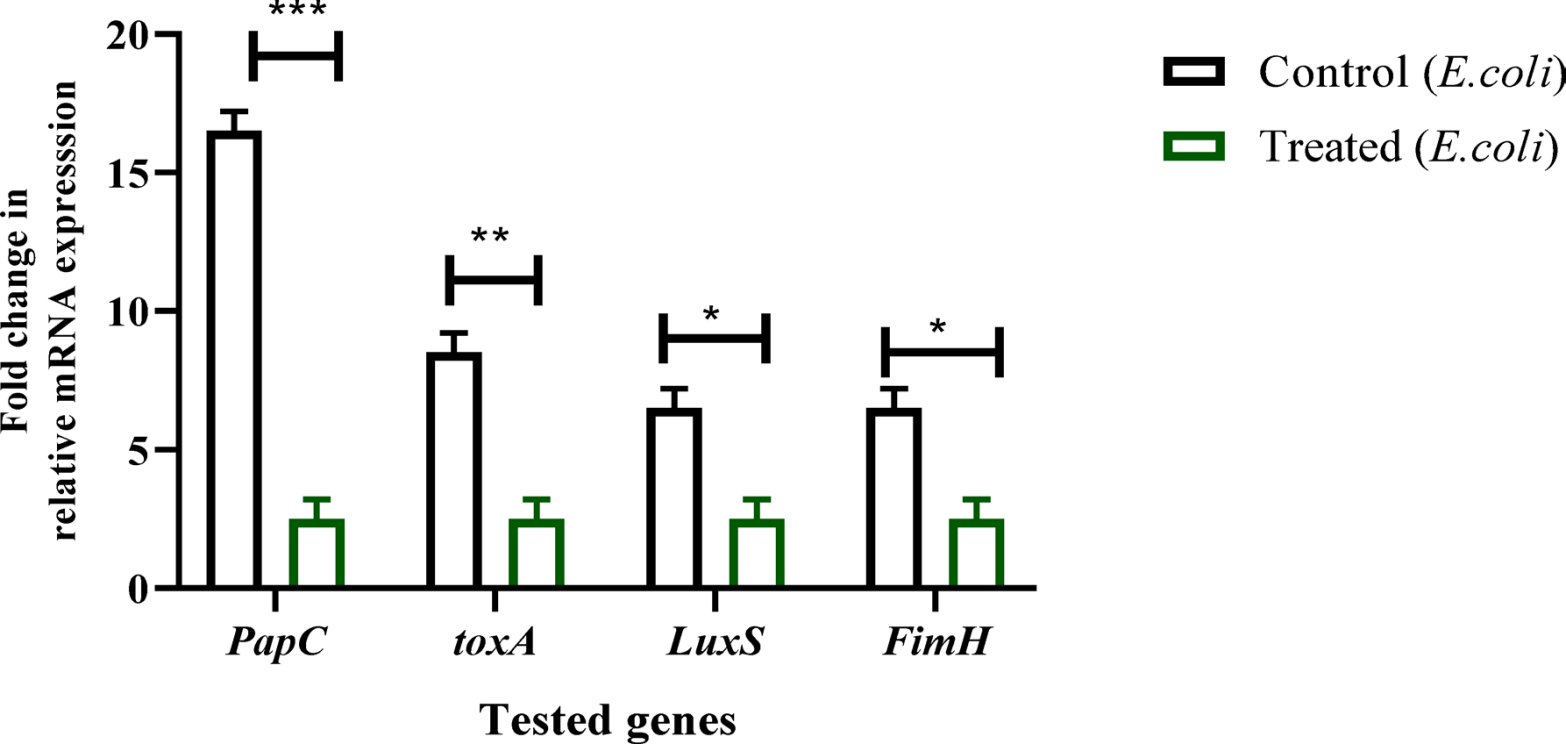
Gene expression outcomes for vilrulence genes in *E. coli* treated with CuO/ZnO nanocompsite relted to untreated bacteria (Outcomes were illustrated as means ± SD; *** *P*≤0.001;** *P*≤0.01; * *P*≤0.05).

Quantitative RT-PCR analysis revealed that the expression of quorum-sensing and virulence genes in *E. coli* was significantly suppressed following treatment with the CuO/ZnO nanocomposite. Among these, the adhesion-related gene *fimH* showed the highest downregulation (8-fold, *P* ≤ 0.001). Similarly, the quorum-sensing regulator (*LuxS*) presented decreased by 4-fold (*P* ≤ 0.01). The toxin-associated gene *toxA* and outer-membrane adhesion gene *PapC* both revealed 3-fold down-regulated (*P* ≤ 0.05) when compared to control. These all point to the broad inhibitory action of the CuO/ZnO nanocomposite to *E. coli* virulence determinants, which may eventually limit adhesion, toxin production, and bacterial cellular communication (Fig. 10). The strong inhibition of *fimH* and *luxS* demonstrates the nanocomposite’s ability to reduce pathogenicity beyond simple bactericidal properties. The *fimH* gene encodes a crucial adhesin that is involved in fimbrial adhesion to host surfaces; downregulation of this gene is anticipated to limit bacterial colonization and biofilm formation. Similarly, *luxS* is essential for the synthesis of autoinducer-2 (AI-2), a universal quorum-sensing molecule responsible for interspecies interaction. Suppressing *luxS* expression may affect population-level connection and virulence regulation. These findings demonstrate that the CuO/ZnO nanocomposite exerts anti-virulence effects beyond simple bactericidal action, offering a promising approach to reduce infection severity and limit the emergence of antimicrobial resistance^55,56^.

To better contextualize the antibacterial efficiency of the biosynthesized CuO/ZnO nanocomposite, a comparison with previously reported metal and metal oxide nanoparticles is presented in (Table 3). The comparison highlights differences in particle composition, reported MIC values, and antibacterial mechanisms. Notably, the CuO/ZnO nanocomposite developed in the present study demonstrated competitive antibacterial performance, combined with significant anti-virulence activity, which is not commonly evaluated in many nanoparticle studies.

**Table 3:** Comparative antibacterial efficiency of different nanoparticles against MDR *E. coli*.

Nanoparticle Type	Target Organism	Reported MIC (µg/mL)	Key Mechanism	Reference
CuO nanoparticles	MDR *E. coli*	125–250	ROS generation, membrane damage	^21^
ZnO nanoparticles	MDR *E. coli*	100–200	Zn²⁺ release, oxidative stress	^19^
Ag nanoparticles	MDR *E. coli*	10–50	DNA interaction, protein denaturation	^49^
CuO/ZnO nanocomposite	*E. coli*	75–150	Heterojunction ROS enhancement	^24^
CuO/ZnO nanocomposite (Present Study)	Clinical MDR *E. coli* (PX674707)	62.5 ± 0.2	ROS generation, membrane disruption, quorum sensing & virulence gene suppression	Present study

## Conclusion

In conclusion, this study reports the successful eco-friendly mycosynthesis of a CuO/ZnO nanocomposite using *A. terreus*. The nanocomposite exhibited controlled nanoscale morphology, high crystallinity, and potent antibacterial activity against a clinically isolated multidrug-resistant *E. coli* strain. The nanocomposite exerted a dual mode of action involving reactive oxygen species generation, membrane disruption, metal ion release, and significant downregulation of quorum-sensing and virulence-associated genes (*fimH*, *papC*, *toxA*, and *luxS*). These findings provide mechanistic evidence that green-synthesized CuO/ZnO nanocomposites can function not only as bactericidal agents but also as anti-virulence modulators. Therefore, future studies should focus on in vivo validation, detailed cytotoxicity assessment, biofilm inhibition analysis, and scalable production optimization to facilitate clinical translation and potential integration into antimicrobial coatings, wound dressings, and biomedical device applications.

## Supplementary Information

Below is the link to the electronic supplementary material.

Supplementary Material 1

## Data Availability

The datasets used and/or analyzed during the current study available from the corresponding author on reasonable request. The isolated and identified MDR E. coli was already identified using 16 S RNA, and a sequence was added to NCBI gene bank with accession number (PX674707) (https://www.ncbi.nlm.nih.gov/nuccore/PX674707).

## Abbreviations

MDR: Multidrug-resistant
HPLC: High performance liquid chromatography
GC-MS: Gas chromatography- mass spectrometry
TEM: Transmission electron microscope
FTIR: Fourier transform infrared spectroscopy
XRD: X-ray diffraction
qRT-PCR: Quantitative reverse transcription PCR
