# Supplementary material for: Green mycosynthesis of a CuO/ZnO heterojunction nanocomposite using Aspergillus terreus and its antibacterial and anti-virulence activity against multidrug-resistant Escherichia coli
Source: Sci Rep. 2026 Apr 13;16:12350. doi: 10.1038/s41598-026-44775-z (PMC13079814; doi:10.1038/s41598-026-44775-z)
Supplement: Supplementary file 1 — Supplementary Material 1 [file 41598_2026_44775_MOESM1_ESM.docx]

**Supplementary Table 1.** Primers used for qRT-PCR analysis of *E. coli* virulence genes

| Gene | Primer Sequence (5′–3′) |
| --- | --- |
| *fimH* | F: TGCAGAACGGATAAGCCGTGG  R: GCAGTCACCTGCCCTCCGGTA |
| *papC* | F: GACGGCTGTACTGCAGGGTGTGGCG  R: ATATCCTTTCTGCAGGGATGCAATA |
| *toxA* | F: GGTAACCTGGGTATGGAAGG  R: GAGGTGATGTTGTTGATGCC |
| *luxS* | F: ATGCCGTTGTTAGATAGCTT  R: TTAGGCAGTTTTCGTCGTTC |
| *16S rRNA* | F: GGTTAAGTCCCGCAACGAGC  R: GGACTACCAGGGTATCTAATCCTGTT |

**Supplementary Table 2.** Time-kill kinetics of CuO/ZnO nanocomposite against MDR *E. coli*#

| Time (h) | Control (log₁₀ CFU/mL) | 1× MIC (log₁₀ CFU/mL) | 2× MIC (log₁₀ CFU/mL) |
| --- | --- | --- | --- |
| 0 | 6.20 ± 0.10 | 6.20 ± 0.08 (ns) | 6.20 ± 0.09 (ns) |
| 2 | 6.80 ± 0.12 | 5.60 ± 0.15 * | 4.10 ± 0.13 *** |
| 4 | 7.30 ± 0.14 | 4.80 ± 0.18 ** | 2.70 ± 0.16 *** |
| 6 | 7.90 ± 0.16 | 4.10 ± 0.17 ** | 2.10 ± 0.14 *** |
| 8 | 8.10 ± 0.18 | 3.50 ± 0.20 *** | 1.80 ± 0.12 *** |
| 24 | 8.40 ± 0.20 | 2.90 ± 0.16 *** | 1.20 ± 0.10 *** |

#Data are expressed as mean ± standard deviation (SD) of three independent experiments (*n* = 3). Statistical analysis was performed using one-way ANOVA followed by Tukey’s post hoc test comparing treated groups to the untreated control at each time point. ns: not significant; **p* < 0.05; ***p* < 0.01; **p* < 0.001. A ≥3 log₁₀ reduction in CFU/mL compared to the initial inoculum indicates bactericidal activity.
